# The development of polymers and fibers: an interview with Stephen Cheng

**DOI:** 10.1093/nsr/nwae357

**Published:** 2024-10-15

**Authors:** Wei Yan

**Affiliations:** State Key Laboratory for Modification of Chemical Fibers and Polymer Materials, College of Materials Science and Engineering, Donghua University, China

## Abstract

*Polymer and fiber science has evolved significantly since the 1920s, with polymers becoming integral to both industry and daily life. China's fiber research, initiated in the 1950s, has made substantial societal and technological contributions, particularly in chemical fibers. How will future advancements in polymers and fibers address ongoing challenges and drive further innovation*?

*NSR spoke to Prof. Stephen Cheng, a member of the National Academy of Engineering (U.S.) and former Dean of the College of Polymer Science and Polymer Engineering at the University of Akron. He is currently Dean and Honorable Professor at South China University of Technology. His research interests center on the condensed states in polymers, liquid crystals, surfactants and hybrid materials, focusing on the interactions, responses, dynamics and structures of materials at varying lengths, energy and timescales, in which the material itself embodies the technology*.

***NSR*:** What is the current state of polymer science?

***Cheng*:** In 1920, Staudinger presented the Macromolecular (Polymer) Hypothesis in his article ‘Über Polymerisation’ (translated as ‘About Polymerization’ in English [1]), in which, for the first time, the chain paradigm of macromolecules was proposed. Since then, a whole century has passed. Over the course of those 100 years, polymers have essentially been central parts of every industry and technology, and transformed every part of human life. ‘The development of polymerization is perhaps the biggest thing *chemistry* has done, where it has had the biggest impact on everyday life,’ as Lord Todd said [2]. The polymer industry has continuously grown at a rapid pace, reaching a global market size of >$721 billion in 2023.

With the success of industrial developments, nowadays, as a branch of science, we have gained a reasonably good understanding of the nature of these long-chain molecules: their chemical formula, compositions, sequences, topologies, sizes, physical structures and molecular packing, as well as their dynamics in different length scales. In recent decades, there has been a trend to expand the scope of macromolecules beyond the traditional linear macromolecular definition and to seek a broader view based on molecular size [3] and interactions [4]. This is because size and interaction effects confer distinct properties and complexity on macromolecules compared with small molecules.

***NSR*:** How has fiber research in China impacted society, and how will it continue to do so?

***Cheng*:** In chemical fibers, industrialization in Western countries began in the first half of the past century. The first commercialized industry product was Nylon 6,6. By the middle of last century, polymer fibers had become major industries in chemical businesses. In China, chemical fibers were developed much later, in the early 1950s. The country proposed the development of chemical fibers to resolve the contradiction of ‘cotton and grain competition for farmland’ and to address the problem of people's clothing needs. Profs. Baojun Qian and Bairong Fang, who were well-known polymer scientists in China at that time, focused on the urgent needs of the country and proposed to establish

**Figure 1:**
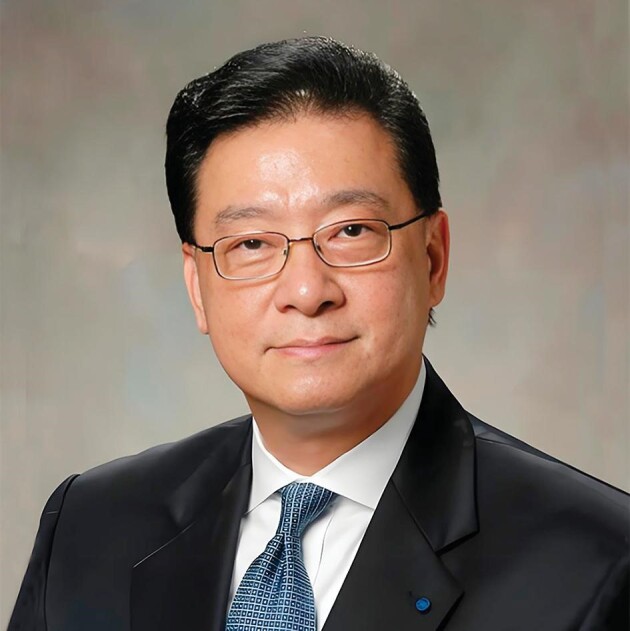
Professor Stephen Cheng (*Courtesy of Stephen Cheng*).

Advances in polymer and fiber sciences are leading to significant innovations in fiber technology, and research and developments of advanced fibers will usher in new opportunities in polymer and fiber sciences.

—Stephen Cheng

China's first undergraduate academic major in chemical fibers at East China Textile Institute (now Donghua University) in 1954, and initiated the development of China's chemical fiber discipline. Over the past 70 years, the chemical fiber discipline has graduated thousands of undergraduate and graduate students, and has developed numerous engineering and technological innovations in fiber industrial production, which have not only met people's daily needs, but also led to high technology applications in many critical fields. According to data released by the China Chemical Fiber Industry Association, China's chemical fiber production reached 68.72 million tons in 2023, accounting for >70% of global production. The export volume was 6.5073 million tons, setting a new historical high.

Chemical fiber is one of the special branches of polymer science and technology. From a dimension point of view, fibers are one-dimensional polymers. The chain nature of polymers provides the structural integrity and performance characteristics of fibers, such as strength, flexibility and thermal stability, which are critically associated with molecular and supermolecular structures and dynamics. These characteristics define fiber science and technology. Advances in polymer and fiber sciences are leading to significant innovations in fiber technology, and research and developments of advanced fibers will usher in new opportunities in polymer and fiber sciences. In this growth area, we can envision chemical fibers combined with Artificial Intelligence, nanotechnology, chemical biology and others to create new materials that can be used to prepare highly functional smart fiber devices with self-repair, environmental response and/or biosensing properties. In addition, applications of advanced manufacturing technologies can also produce fibers with complex structures and specifically designed properties.

***NSR*:** How can the challenges in the fields of polymers and fibers be addressed?

***Cheng*:** Every scientific field has its own birth and growth phases before reaching maturity. One of my best friends, Dr. Andrew Lovinger, who is a member of the National Academy of Engineering of the USA, once said to me that ‘frequently, in science, something new and revolutionary may become so successful that it ends up being taken for granted’ [5]. This is what has happened to the field of polymers, especially in fibers, which had its ‘big bang’ just half a century ago with discoveries of every aspect of fibers ranging from various advanced polymer syntheses, polymer solutions, solid states of crystalline polymers, polymer glasses, polymer blends and copolymers. On the other hand, industrial technologies and applications of polymer fibers had been substantially developed and extended to virtually every area of high technology and our daily lives, covering many orders of magnitude of length scales.

‘Yet, this “big bang” has turned into more of a “whimper” (in the words of T.S. Eliot). It is not that polymers and fibers are no longer important. Polymers and fibers have simply become so pervasive that we just take them for granted.’ Polymer and fiber researchers have moved on to newer and fancier areas that are currently appealing (such as hybrids, nano-everything, bio-…). ‘The question is whether the major problems in polymers and fibers have been solved. Has it been time to move on? Our answer is no, not by a long shot’ [5].

How to tackle these problems in polymers and fibers? Note that these research problems are mostly more difficult than those that have previously been solved. The critically important approach must include three essential aspects. First, our research must not follow traditional ways; instead, we must think out of the box. Only this way of thinking may lead us to achieve scientific discoveries, technological creativities and industrial innovations. Second, any new approach must be interdisciplinary and collaborate with experts in different areas such as physics, chemistry, bioscience and engineering, etc. This has led to the emergence of functional and smart fibers, which hold great potential for addressing challenges in a variety of areas. Nowadays, without these collaborations, the achievement of significant progress and impact in advancing sciences and technology is almost impossible. Third, the majority of research in polymers and fibers has, in principle, deviated from pure fundamental basic research. It is critically associated with technological developments. This requires closely joint research activities between academic research and industrial applications. The aspect of processing engineering must stem from the research and development of industrial laboratories.

The importance of integrity in research practices cannot be overstated. The bottom line in our scientific community is to uphold ethical standards and ensure reproducibility in order to establish credibility. Resilience is a special characteristic that a scientist must possess in order to pursue a commitment of life-long learning and to stay at the frontier of the field in polymer science and fiber technology, continuously generating scientific and practical impacts.

The bottom line in our scientific community is to uphold ethical standards and ensure reproducibility in order to establish credibility.

— Stephen Cheng

## Notes

Wei Yan is a professor at the State Key Laboratory for Modification of Chemical Fibers and Polymer Materials, College of Materials Science and Engineering, Donghua University, China.
